# Molecules that target nucleophosmin for cancer treatment: an update

**DOI:** 10.18632/oncotarget.8599

**Published:** 2016-04-05

**Authors:** Adele Di Matteo, Mimma Franceschini, Sara Chiarella, Serena Rocchio, Carlo Travaglini-Allocatelli, Luca Federici

**Affiliations:** ^1^ Istituto di Biologia e Patologia Molecolari, Consiglio Nazionale delle Ricerche, Rome, Italy; ^2^ Dipartimento di Scienze Mediche, Orali e Biotecnologiche, Università di Chieti “G. d'Annunzio”, Chieti, Italy; ^3^ Ce.S.I.-MeT Centro Scienze dell'Invecchiamento-Medicina Traslazionale, Università di Chieti “G. d'Annunzio”, Chieti, Italy; ^4^ Dipartimento di Scienze Biochimiche “A. Rossi Fanelli”, Università di Roma “Sapienza”, Rome, Italy

**Keywords:** nucleophosmin, B23, acute myeloid leukemia, solid tumours, targeted therapy

## Abstract

Nucleophosmin is a highly and ubiquitously expressed protein, mainly localized in nucleoli but able to shuttle between nucleus and cytoplasm. Nucleophosmin plays crucial roles in ribosome maturation and export, centrosome duplication, cell cycle progression, histone assembly and response to a variety of stress stimuli. Much interest in this protein has arisen in the past ten years, since the discovery of heterozygous mutations in the terminal exon of the *NPM1* gene, which are the most frequent genetic alteration in acute myeloid leukemia. Nucleophosmin is also frequently overexpressed in solid tumours and, in many cases, its overexpression correlates with mitotic index and metastatization. Therefore it is considered as a promising target for the treatment of both haematologic and solid malignancies. NPM1 targeting molecules may suppress different functions of the protein, interfere with its subcellular localization, with its oligomerization properties or drive its degradation. In the recent years, several such molecules have been described and here we review what is currently known about them, their interaction with nucleophosmin and the mechanistic basis of their toxicity. Collectively, these molecules exemplify a number of different strategies that can be adopted to target nucleophosmin and we summarize them at the end of the review.

## INTRODUCTION

**Figure 1:**
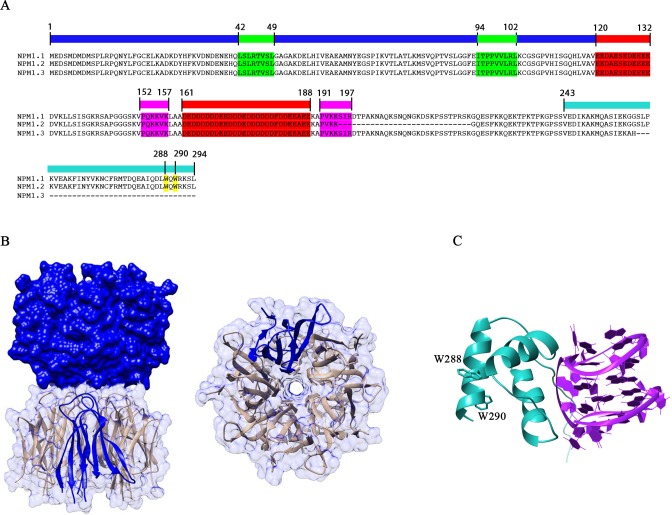
Domain organization and structure of NPM1 **A.** Primary structures of NPM1.1, NPM1.2 and NPM1.3 are shown. The blue bar marks the N-terminal core domain. Nuclear export signals (NES) in the N-terminal domain are highlighted in green. Acidic-rich regions in the central domain are highlighted in red, while the bipartite nuclear localization signal (NLS) is highlighted in magenta. The cyan bar marks the C-terminal nucleic acid binding domain. Trp288 and Trp290, which form the nucleolar localization signal (NoLS) are highlighted in yellow. Notably NPM1.2 lacks part of the NLS while NPM1.3 lacks most of the C-terminal domain and its NoLS. **B.** Crystal structure of the N-terminal core domain [32]. Five monomers associate to form a pentameric assembly and two pentamers interact in a head-to-head fashion to generate a decamer. **C.** The structure of the C-terminal domain of NPM1 (in cyan) is shown in complex with a G-quadruplex sequence from the *c-MYC* promoter (in magenta) [43]. Trp288 and Trp290 side chains are also highlighted.

Nucleophosmin (also known as NPM1, B23, No38, numatrin) is a phosphoprotein, mainly localized at nucleoli [1]. The *NPM1* gene maps to chromosome 5q35 and is expressed in three isoforms through alternative splicing (Figure 1A). Isoform NPM1.1 (P06748-1) (294 residues) is the most abundant one and displays nucleolar localization. Isoform NPM1.2 (P06748-2) lacks an in-frame exon (exon 8) resulting in a shorter protein with respect to NPM1.1 in which an internal segment (residues 195-223) is lacking. The third isoform, NPM1.3 (formerly known as B23.2; P06748-3), uses an alternative exon at the 3′ end, which is responsible for a shorter protein construct lacking the last 35 aminoacids with respect to NPM1.1 [2]. This isoform is expressed to low levels and has nucleoplasmic localization. The most abundant NPM1.1 isoform, which will be called NPM1 from now on, is expressed in all tissues. All studies we report here are focused on this isoform.

NPM1 is one of the most abundant proteins in the granular region of nucleoli; it plays a crucial role in maintaining nucleolar structure and is therefore considered one of the “hub” proteins of the nucleolus [3, 4]. NPM1 is involved in many and different cellular functions which have been extensively reviewed recently [5, 6, 7]. Among them NPM1 plays a role in: i) rRNA expression and maturation [8, 9], ii) ribosome assembly and export [10, 11], iii) centrosome duplication [12, 13], iv) DNA replication, recombination, transcription, and repair [1, 5, 6, 14, 15], v) molecular chaperoning for histones and other proteins [5, 16, 17, 18]. Many of these functions are fulfilled through the interaction with different protein partners and indeed NPM1 has been reported to interact with a plethora of proteins [reviewed in 6]. Of particular importance here is the involvement of NPM1 in the p14ARF-HDM2-p53 signaling axis. NPM1 has been reported to interact with all these three proteins in different cellular contexts [19, 20, 21]. In particular NPM1 appears to be the major cellular interactor of p14ARF [22] and to be responsible for p14ARF nucleolar localization in unstressed cells. p14ARF mutants failing to interact with NPM1 are highly unstable and display low anti-proliferative activity [23]. Moreover, overexpressed NPM1 directly interacts with c-MYC and controls c-MYC induced hyperproliferation and transformation activities [24], while being also essential for c-MYC nucleolar localization and c-MYC mediated rRNA transcription [25].

Various aspects of the NPM1 structure, trafficking and post-translational modifications are central to its pleiotropic behavior. NPM1 displays a modular organization in distinct domains each endowed with specific activities (see below). Furthermore, NPM1 mainly resides in nucleoli but can shuttle between nucleoli, nucleoplasm, and cytoplasm thanks to its different localization signals. NPM1 cellular localization during the various phases of the cell cycle or in response to stress signals, as well as the interactions established by NPM1, are all tightly regulated through post-translational modifications. Indeed NPM1 phosphorylation, acetylation, sumoylation, ADP ribosylation and poly-ubiquitination have all been reported [reviewed in 5].

## NPM1 STRUCTURE

NPM1 shows a modular organization in which three distinct regions can be envisaged: i) the N-terminal region, often referred to as the “core” domain, is mainly responsible for the chaperone activities and for the interaction with protein partners [26, 27, 28]. This region contains two nuclear export signals (NES) (Figure 1A); ii) a central region, predicted to be natively unstructured, which contains a bipartite nuclear localization signal (NLS) (Figure 1A); iii) the C-terminal region of the protein is important for the interactions with nucleic acids [27, 29], contains the nucleolar localization signal (NoLS) (Figure 1A), and is the region where AML-associated mutations occurr [30].

The NPM1 N-terminal core domain shows high similarity to proteins belonging to the nucleoplasmin family [31]. The three-dimensional structures of this domain from human [32] and mouse [28] NPM1 as well as from the homologous Drosophila dNLP [33] and Xenopus NO38 [34] proteins have been determined. They all consist of eight antiparallel β-strands forming a β-barrel with jelly-roll topology. Five monomers tightly associate to form a crown-shaped pentamer (Figure 1B) and two pentamers interact in a head-to-head fashion to form a decamer, with few contacts between the two pentamers (Figure 1B). Whether this decameric assembly is physiologically relevant is presently unknown. NPM1 functions and cellular localization have been reported to be correlated with its oligomerization state [5, 35, 36]. In all determined structures, the pentameric assembly is stabilized by extensive hydrophobic and hydrogen-bonding interactions, ensuring for this domain a high thermal and chemical stability, typical of molecular chaperones [37]. Experimental evidences suggest that ionic strength, divalent ions and interactions with protein partners also contribute to stabilize the pentameric organization of NPM1 [28, 38]. However, a key and opposed role appears to be played by phosphorylation of serine and threonine residues by several different kinases [28]. The sequential phosphorylation of such residues, first those solvent exposed and then those buried at the monomer-monomer interface, greatly destabilizes the oligomeric state of NPM1 promoting monomerization [28]. Monomers are in turn intrinsically unstable and, following pentamer dissociation, completely unfold [28].

The N-terminal core domain is followed by a poorly characterized central region, predicted to be natively unstructured. This region contains two long acidic stretches, spanning residues 120-132 and 161-188 respectively. They are composed of several consecutive glutammate or aspartate residues (Figure 1A) and thought to be important, in cooperation with the N-terminal domain, for the histone chaperoning activity played by NPM1 [31]. Before and after the second acidic stretch, a bipartite nuclear localization signal (NLS) is present (residues 152-157 and 191-197). The terminal part of the region is instead markedly basic and may cooperate with the C-terminal domain in shaping its binding properties. Interestingly, a protein segment comprising the second acidic stretch, the terminal basic region and part of the C-terminal domain (residues 140-259) has been associated to a ribonuclease activity on the internal transcribed segment 2 (ITS2) of 47S ribosomal pre-mRNA, essential for ribosome maturation [10].

The C-terminal domain of NPM1 consists of a three-helix bundle (Figure 1C) [39], stabilized by a set of strictly conserved aromatic residues (Phe268, Phe276, Trp288, and Trp290) [7]. This domain contains the NPM1 nucleolar localization signal (NoLS), encompassing the two tryptophan residues Trp288 and Trp290. This aromatic-rich NoLS is very atypical and, to our knowledge, present only in NPM1. Mutations of one or both Trp288 and Trp290 residues cause unfolding of the three-helix bundle and loss of NPM1 nucleolar localization [39, 40]. Such mutations are typical of AML patients, as it will be detailed later.

The DNA and RNA binding activity of NPM1 is exerted by its C-terminal domain. This was initially shown to have a preference for single stranded DNA and RNA over duplex DNA, however no sequence requirements for nucleic acid binding were uncovered [27, 41]. More recently, NPM1 was shown to specifically target a G-rich sequence at the *SOD2* gene promoter [42] which adopts a G-quadruplex structure under physiological conditions [29]. The structure of NPM1 C-terminal domain in complex with a G-quadruplex oligonucleotide derived from the *c-MYC* promoter was also investigated [43]. It was shown that the three-helix bundle engages the G-quadruplex phosphate scaffold with a positively charged groove located between helices H1 and H2 (Figure 1C). Structural studies also revealed that the terminal part of the central domain, which is unstructured and markedly positively charged, is also necessary for high affinity binding, through both long range electrostatic effects and transient interactions with the G-quadruplex [44, 45]. NPM1 loses its nucleolar localization following lysine acetylation played by p300 [46] and, consistently with structural studies, both lysine residues located in the three-helix bundle at the G-quadruplex interface (Lys250, Lys257 and Lys267) [43] and lysine residues located in the flanking unstructured tail (Lys229 and Lys230) are acetylated by p300 [46].

## NPM1 AND CANCER

NPM1 is over-expressed in a variety of solid tumours of different histological origin including prostate [47], liver [48], thyroid [49], colon [50], gastric [51], pancreas [52], glioma and glioblastoma [53, 54], astrocytoma [55] and others. NPM1 overexpression often correlates with mitotic index and metastatization and it has been proposed as an adverse prognostic marker in a number of such malignancies [5, 56]. Even though amplification of the *NPM1* locus has never been shown, the *NPM1* gene is a target of the oncogenic c-MYC, which stimulates NPM1 expression by direct binding to the *NPM1* promoter [57].

The specific contribution of overexpressed NPM1 to cancer development is not fully understood but it may arise from multiple factors. First, NPM1 overexpression is correlated to increased ribosome biogenesis and protein synthesis and both these functions are amplified in tumour cells [58]. Furthermore, NPM1 plays a role in stimulating DNA repair following oncogene activation and reduces apoptotic or senescence response [5, 6, 59]. Accordingly, a model has been recently proposed [5] whereas, when oncogene activation arises in a normal cell as a first genetic event, overexpression of NPM1 may contribute to reinforce the DNA damage response thus keeping DNA damage and the consequent genomic instability to a level that the cell can sustain. This would in turn allow cells to select for the cooperative mutations necessary for transformation.

NPM1 is also heavily implicated in haematological malignancies, being its gene both the target of different chromosomal translocations or of frequent mutations. In 30% of anaplastic large cell lymphoma (ALCL) patients a t(2;5) translocation fuses the 5′ end of NPM1 gene with the 3′ portion of the ALK (anaplastic limphoma kinase) gene. This leads to the expression, in the cytoplasm of cancer cells, of a chimeric protein consisting of the NPM1 N-terminal oligomerization domain fused to the ALK tyrosine kinase domain [60, 61]. This chimera is a major driver in ALCL tumourigenesis [62] and the role of the NPM1 moiety is thought to be that of facilitating, through its oligomerization, the dimerization and thus the constitutive activation of the ALK tyrosine kinase domain.

A second rare event was found in acute promyelocytic leukemia (APL) patients. Here, as a consequence of a t(5;17)(q35;q31) translocation, the NPM1 N-terminal domain is fused to the DNA-binding domain of retinoic acid receptor α (RARα). Also in this case, and similarly to the most common PML-RARα chimera, NPM1 facilitates dimerization of the RARα moiety thus interfering with the RARα transcriptional activity [63]. The resulting arrest of myeloid differentiation may be reversed by treatment with all-trans retinoic acid (ATRA) [64].

A third chromosomal translocation at t(3;5)(q25;q35) has been identified in a small subgroup (less than 1%) of acute myeloid leukemia (AML) patients. This event generates a protein chimera comprising the first 175 aminoacids of NPM1 (the N-terminal domain plus a portion of the central unstructured region) and the entire coding sequence of the protein MLF1 (myelodisplasia/myeloid leukemia factor 1) [65]. The role of this chimera in tumourigenesis has not been fully elucidated.

Beside the specific role played by the NPM1 moiety in the different chimeras, in all cases haploinsufficiency for the *NPM1* WT gene is generated and partial dislocation of the NPM1 WT protein in the cytosol is observed, as a consequence of heteroligomerization with protein chimeras. Interestingly, NPM1 haploinsufficiency is also observed in myelodisplastic syndromes with 5q deletion [66], suggesting that it may confer *per se* a proliferative advantage in the myeloid lineage.

In 2005 the *NPM1* gene was identified as the most frequently mutated one in AML, accounting for around 60% of patients with normal kariotype and 35% of total cases [30]. Mutations map to the last exon of the gene and are always heterozygous [67, 68]. More than 30 different mutations have been identified but the consequences at the protein level are similar in all cases: due to duplication or insertion of short nucleotide stretches at exon XII of the NPM1 gene, the reading frame is altered leading to a protein that has acquired four additional residues at the C-terminus and has a different sequence in the last seven residues. Both triptophans 288 and 290, or only Trp288 in some unfrequent mutants, which constitute the nucleolar localization signal (NoLS), are replaced and the whole C-terminal domain of the protein is totally unfolded or largely destabilized [39, 69, 70, 71]. Furthermore, the newly generated sequence forms a novel NES which reinforces the two already present at the N-terminal region of the protein. Disruption of the NoLS and appearance of a new NES account for the aberrant cytoplasmic localization of mutated NPM1 [40], which is the hallmark of this kind of leukemia (hence mutated NPM1 is also termed NPM1c+, from cytoplasmic positive). Furthermore, since NPM1c+ oligomerizes with the wild-type protein produced by the normal allele, through its unaltered N-terminal domain, the majority of wild-type NPM1 is also translocated in the cytosol and only a small fraction still resides in the nucleoli of leukemic blasts. A wealth of different data [72, 73] suggests that NPM1 mutations act as a founder genetic lesion in this kind of leukemia and therefore AML with NPM1 mutation has been included as a new provisional entity in the WHO 2008 classification of myeloid neoplasms [74]. AML with mutated *NPM1* may be further stratified into two different categories: those patients where concomitant *FLT3-ITD* (FMS-like tyrosine kinase internal tandem duplication) is absent, usually respond to standard induction therapy and have favourable prognosis; when *FLT3-ITD* sums up to *NPM1* mutations (around 30% of cases) the prognosis is much worse. In all cases relapse is frequent and *NPM1* mutations are typically present at relapse [75].

The exact mechanism through which NPM1c+ exerts its transforming activity is not yet fully understood, but all evidences point to the hypothesis that “placing a critical regulator at the wrong place in the wrong time” may be the driving force [76]. In particular NPM1c+ may confer a proliferative advantage to blasts through the gain of several and unwanted “new” cytosolic functions. A number of them have been elucidated. For instance NPM1c+ interacts, through its unchanged N-terminal domain, with the tumour suppressors p14ARF and Fbw7γ, the major E3-ubiquin ligase targeting c-MYC, which are both translocated in the cytosol and there proteasomally degraded [77, 78]. Therefore, an important tumour suppressor pathway is hampered while, at the same time, an oncogene product like c-MYC is stabilized. Also, NPM1c+ binds and inhibits caspases 6 and 8, thus directly impacting on the execution of apoptosis [79]. Furthermore, NPM1c+ binds and inhibits the PTEN deubiquitinating enzyme HAUSP, resulting in PTEN cytoplasmic polyubiquitinilation and degradation [80]. Thus a third important tumour suppressor is also deregulated by NPM1c+ [80]. These cytosolic activities promote leukemic blasts survival and may also counteract the nucleolar stress caused by NPM1 delocalization. Additionally, the unfolded C-terminal domain of NPM1c+ has been recently shown to contain amyloidogenic regions that may also contribute to the gain of cytosolic functions played by the protein [81].

### Targeting NPM1 for cancer treatment

A comprehensive description of the pleiotropic functions played by NPM1 and its role in cancer has been the subject of a number of excellent reviews to which we refer the reader [1, 5, 6, 7, 71, 72]. A common conclusion in all these analyses is that NPM1 should be considered as a target for the treatment of several tumours, noticeably haematological malignancies where the *NPM1* gene is mutated or found at the junction of chromosomal translocations, but also solid tumours where the gene is overexpressed.

Interestingly, over the course of the last 10 years, several molecules that target NPM1 have been indeed discovered and their effect and therapeutic potential has been investigated to various extent. Therefore we thought that it might have been timely and appropriate to review here what is currently known about these molecules, their interaction with NPM1 and the mechanistic basis for their toxicity.

## NSC348884

**Figure 2:**
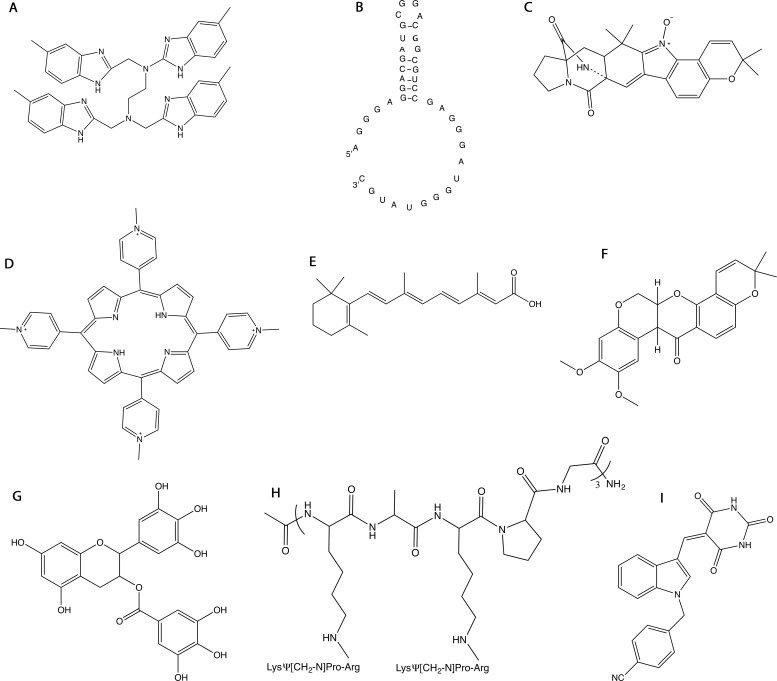
Structures of molecules that target NPM1 **A.** NSC348884. **B.** 1A1(1-40) RNA aptamer. **C.** (+)-avrainvillamide. **D.** TmPyP4. **E.** All trans-retinoic acid (ATRA). **F.** deguelin. **G.** (−)-epigallocatechin-3-gallate (ECGT). **H.** NucAnt 6L. **I.** YTR107.

**Figure 3:**
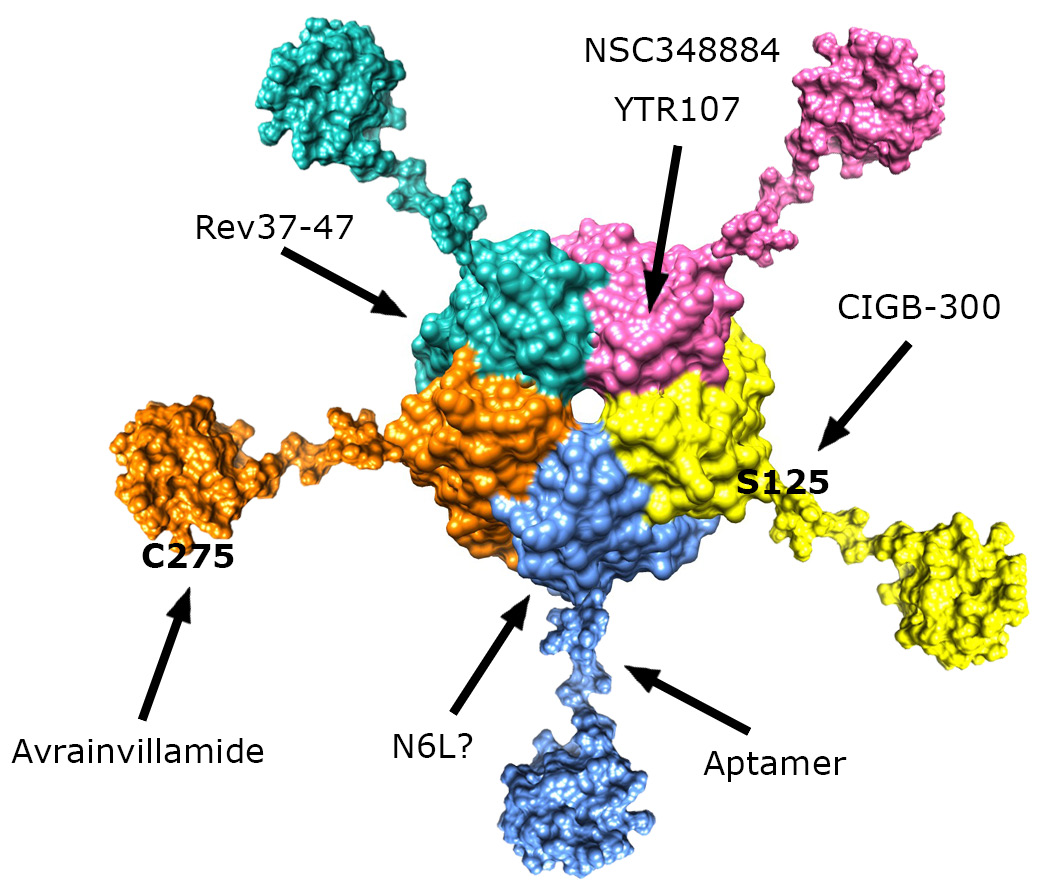
Schematic representation of full-length NPM1 structure showing the pentamer formed by the N-terminal domains, followed by the central unstructured regions and culminating with the folded C-terminal domains The sites recognized by NPM1-interacting molecules are indicated, when known.

NPM1 oligomerizes through interactions mediated by its N-terminal domain. Loss of oligomerization has been shown to promote unfolding of the domain [28], suggesting a consequent impairment of its functions. The hypothesis of targeting the oligomerization properties of NPM1 to interfere with its functions, including its antiapoptotic activities, led to the identification of NSC348884, the first small molecule inhibitor reported to specifically interact with NPM1 [82]. A pharmacophore hypothesis was devised from the analysis of the hydrophobic interface between monomers in the pentameric ring and used to screen “in silico” a large library of compounds. NSC348884 ((di-[((6-methyl-1H -benzo[d]imidazol-2-yl)methyl)((5-methyl-3-oxo-3H -indol-2-yl)methyl)]) aminoethane) (Figure 2A and Figure 3) was the best hit and used for subsequent functional studies. NSC348884 was initially shown to promote monomerization of NPM1 in LNCaP (androgen-sensitive prostate adenocarcinoma) and HCT116 (colorectal carcinoma) cell lines, in which NPM1 is wild-type and highly expressed. Cell viability assays, conducted on LNCaP and Granta (mantle cell lymphoma) cells showed cellular toxicity with IC_50_ varying in the 1.5-4 micromolar range (Table 1). Subsequent experiments demonstrated a remarkable synergic effect of NSC348884 with doxorubicin. The combined used of sub-cytotoxic doses of both drugs led to a complete loss of cell viability [82]. These promising data led to the investigation of the mechanistic basis of cellular toxicity. First, it was shown that NSC348884 counteracts the anti-apoptotic activity of over-expressed NPM1 and promotes apoptosis in LNCaP and Granta cells in a dose-dependent fashion, as seen both by morphologic analysis and annexin V staining. It is well known that NPM1 knockdown results in increased levels of p53 and of its phosphorylation at the Ser15 site [83]. Similarly, NSC348884 treatment was shown to exert both effects and also to elevate levels of p21, a key transcriptional target of p53. NPM1 is known to interact with the tumour suppressor p14ARF and to sequester it in nucleoli. When moving to the nucleoplasm, p14ARF interacts with HDM2, the E3-ubiquitin ligase for p53, resulting in elevated p53 levels [84]. NPM1 has also been shown to directly interact with p53 and prevent its phosphorylation at Ser15 [83]. Both of these NPM1 antiapoptotic activities may be compromised by treatment with NSC348884 thus explaining its apoptotic effect in cells overexpressing NPM1. Similar results, i.e. NPM1 monomerization and cell growth inhibition, were also recently shown in the hepatic carcinoma HepG2 cells treated with NSC348884 [85].

**Table 1:** Molecules that interact with NPM1

Molecule	Efficacy	Target Cells	Clinical Trails	Site of interaction	Key ref.
NSC34884	IC_50_ between 1,4-4,0 μM	LNCaP (prostate adenocarcinoma) HCT116 (colorectal carcinoma) Granta (mantle cell lymphoma) HepG2 (hepatocellular carcinoma) OCI-AML3 (AML with NPM1c+)		oligomerization surface of the N-terminal domain	82,85,86
Rev37-47	GI_50_=95,1 μM	Ras-3T3 (Ras transformed mouse fibroblasts)		N-terminal domain	91
RNA aptamers 1A1 and 1A1(40)	K_D_= 30-33 nM (IC_50_ or GI_50_ not available)	HeLa (cervical carcinoma) MCF-7 (breast cancer) SGC7901 (gastric cancer)		Central Region (114-187)	94
CIGB-300	IC_50_ between 20-180 μM	H-125 (non-small cell lung) H-82 (small cell lung cancer) HeLa (cervical carcinoma) Jurkat (T-cell leukemia) MCF-7 (breast cancer) Hep-2 (laryngeal carcinoma) TC-1 (murine lung carcinoma)	Phase I (locally advanced cervical cancer) Phase II (squamous cell carcinoma or cervix adeno carcinoma)	Binds Ser125 and inhibits phosphorilation by CKII	98,99 106,107
Avrainvillamide	GI_50_ between 0,33-0,52 μM	T-47D (breast carinoma) LnCaP (prostate adenocarcinoma) OCI-AML2 (AML with wtNPM1) OCI-AML3 (AMl with NPM1c+)		C-terminal domain, alkylates Cys275	108,109 110
TmPyP4	IC_50_=0,2 μM (with halogen (irradiation at 7,2 J/cm^2^) GI_50_≈15 μM	B78-H1 (murine melanoma) SW480 (colon carcinoma)		binds rDNA G-quadruplexes recognized by C-terminal domain	113,114 116,117
ATRA/ATO	IC50≈1 μM	OCI-AML3 (AMLwith NPM1c+) IMS-M2 (AMLwith NPM1c+) AML patients primary blasts		promote degradation of NPM1c+ by oxidation of Cys288	120,121
Deguelin	IC_50_=1,49 μM	OCI-AML3 (AMLwith NPM1c+)		promote degradation of NPM1c+ with unknown mechanism	124
EPTG	IC_50_=5-20 μM (according to initial cell density)	IMS-M2 (AMLwith NPM1c+)		promote degradation of NPM1c+ with unknown mechanism	125
NucAnt 6L	GI50=5-38 μM K_D_= 1nM	T29 (murine T-cell lymphoma) MOLT-4 (ALL) Jurkat (T-cell leukemia) A20 (murine B-cell lymphoma) HL-60 (APL) HCT116 (colorectal carcinoma) MDA-MB231(mammary gland Adenocarcinoma) MDA-MB435 (melanoma) U87-MG (glioblastoma) U373-MG (glioblastoma)	Phase I/IIa (multiple solid tumors)	binds full-length NPM1 (individual domains not tested)	126,127
YTR107	not available	HT29 (colorectal carcinoma) D54 (glioblastoma) PANC-1 (pancreatic duct adenocarcinoma)		oligomerization surface of the N-terminal domain	129,130 131

The effect of NSC348884 in AML was analysed by comparing cells expressing NPM1c+ (OCI-AML3) and cells expressing only wild type NPM1 (HL-60 and OCI-AML2) [86]. Interestingly, NSC348883 was found to be more effective in disrupting NPM1 oligomerization in OCI-AML3 cells compared to HL-60 and OCI-AML2 cells. This is somewhat unexpected since mutations target the C-terminal domain of the protein while NSC348884 targets the N-terminal domain. Furthermore, NSC348883 markedly induced higher levels of apoptosis in OCI-AML3 cells than in the NPM1 wt cells. A synergic effect was found in the combined treatment of OCI-AML3 cells or primary AML cells expressing NPM1c+ with NSC348884 and all-*trans*-retinoic acid (ATRA), but not in cells expressing wild-type NPM1 only. However, NSC348883 treatment alone or co-treatment with ATRA was significantly less effective in inducing apoptosis in primary AML cells co-expressing NPM1c+ and FLT3-ITD, consistent with the poorer prognosis of patients carrying both alterations with respect to patient with NPM1c+ only [86].

Overall, these data suggest that molecules targeting NPM1 oligomerization may be effective against both solid malignancies overexpressing NPM1 and in AML with NPM1c+ expression, especially when used in combination with established chemotherapy. This therapeutic strategy may be also attractive for tackling haematological disorders caused by chromosomal translocations in which the N-terminal region of NPM1 is fused to other proteins, i.e. NPM-ALK, NPM-RARα and NPM-MLF1. As already discussed above, in such chimeric oncoproteins the role of the NPM1 moiety is probably that of favouring, through its oligomerization, the dimerization of the fusion partners, which are then constitutively activated. However, NSC348884 has not been yet tested in such tumours.

## REV-NLS

Different studies have suggested that NPM1 behaves as a nucleolar “hub”, favouring the localization and accumulation of its protein partners in the nucleolus thanks to reciprocal interactions [87]. Although extensive biochemical and structural characterization of these interactions is still lacking and many observations available in the literature are conflicting, various studies have identified NPM1 and in particular its N-terminal region as one of the receptors responsible for the nucleolar localization of many proteins (Figure 3) [28].

This topic was initiated by studies on the interaction between NPM1 and the Rev protein from the human immunodeficiency virus-1 (HIV-1). Rev is a protein essential for virus replication and was initially shown to interact with NPM1 [88, 89]. Later on, the Rev sequence recognized by NPM1 was identified and shown to coincide with the highly basic sequence necessary for Rev nuclear/nucleolar localization (i.e. its NoLS) [90]. To analyse the effect played in cancer cells by interfering with NPM1 protein-protein interactions, different Rev peptides were administered to Ras-3T3 cells [91]. In particular, the Rev37-47 peptide (ARRNRRRRWRE), which binds *in vitro* NPM1 with submicromolar affinity [90] was shown to be active with GI_50_=95.1 μM (Table 1). Other Rev-derived peptides with reduced or no affinity for NPM1 had virtually any effect. Rev37-47 also efficiently inhibited colony formation in soft agar, suggesting that the peptide could revert the transformed phenotype of Ras-3T3 cells to a normal phenotype. Further experiments on nude mice inoculated subcutaneously with Ras-3T3 cells confirmed the efficacy of Rev37-47 to consistently reduce tumour growth. Finally Rev37-47 was shown to synergize with doxorubicin in reducing tumour growth in the xenografts. Similarly to what already shown with drugs that influence the oligomeric state of NPM1, treatment with Rev37-47 results in p53 increased levels and transcriptional activity. Consistently, the cytotoxic effect of this Rev peptide was abrogated by siRNA directed against p53 [91].

Overall, though very preliminary, these data suggest that a molecule targeting the NPM1 surface that interacts with HIV Rev, plays a toxic effect on cancer cells. Since NPM1 interacts with many proteins, the protein-protein interaction surface targeted by Rev37-47 may be common to other NPM1 partners (Figure 3). Therefore a single molecule may compromise many of the interactions established by NPM1 at once.

## RNA APTAMERS

Aptamers are small synthetic RNA or single stranded DNA molecules that can bind and inhibit with high affinity and specificity target molecules. Many aptamers are currently in development and have already undergone clinical trials as promising therapeutic tools against different diseases including cancer [92].

With the aim of inhibiting the antiapoptotic activity of NPM1, an effort to select functional aptamers was paid by using the technique known as “systematic evolution of ligands by exponential enrichment” (SELEX) [93]. This procedure, performed against full-length NPM1, led to the identification of the 1A1 RNA aptamer and its truncated 40-mer form 1A1(1-40) (Figure 2B), both able to bind NPM1 with K_D_=33 nM and K_D_=30 nM, respectively (Table 1) [94]. Subsequent efforts were paid to identify the exact NPM1 domain targeted by the aptamer *in vitro*. These studies showed that the isolated NPM1 central region (residues 114-187) was bound with same affinity as full-length NPM1 while the N-terminal (1-113) and C-terminal (188-294) domains were not bound by the aptamer (Figure 3). Further experiments performed *in vitro* with full-length NPM1 demonstrated that the aptamer interferes with NPM1 oligomerization. It is well-known that the isolated N-terminal domain of the protein, which is not bound by the aptamer in isolation, is *per se* sufficient to form very stable pentamers and dimers of pentamers [27, 32, 33]. Therefore, further experiments are necessary to understand the dependence of NPM1 oligomers stability on the interplay among different domains in the context of the whole protein.

The effect of aptamers was assayed in different cancer cell lines including MCF-7 (human breast adenocarcinoma). It was confirmed that aptamers interact with NPM1 also *in vivo* and promote monomer accumulation and oligomer depletion. Importantly, immunofluorescence studies indicated that, upon aptamers expression, NPM1 delocalizes from nucleoli to the nucleoplasm [94]. Since it is known that the nucleolar localization signal is localized at the very C-termini of the protein (Trp288 and Trp290), an area far from the putative aptamers binding site, also this effect awaits for a structural explanation; it is possible that currently unknown interactions between NPM1 domains may be destabilized by aptamer binding and interfere with NPM1 ability to associate with nucleoli. From the cell viability point of view, the expression of the aptamers caused an increase of apoptotic cells, comparable to what seen with siRNA mediated NPM1 down-regulation. A synergistic effect in causing apoptosis with the DNA damaging drugs etoposide and cisplatin was also observed. Mechanistically, it was shown that aptamer expression is followed by p14ARF accumulation in the nucleoplasm, p53 increased levels and p21 expression, similarly to what observed with NSC348883 [82].

## CIGB-300

Many protein kinases are established targets in cancer therapy and several kinase inhibitors already entered the clinic or are undergoing clinical trials. Among the protein kinases that could be targeted one is the Ser/Thr Casein kinase 2 (CK2). In fact, high levels CK2 have been found in different cancer cells [95, 96] especially those which show remarkable resistance to death, being this protein a major player in apoptosis suppression [97].

CIGB-300 is a cyclic peptide fused, at the N-terminus, to a cell-penetrating peptide derived from the HIV Tat protein (GRKKRRQRRRPPQ-β-ala-CWMSPRHLGTC with a disulphide bond between the two cysteine residues). This compound was derived by screening a random cyclic peptide phage library against the HPV-16 E7 oncoprotein site targeted by CK2 for phosphorylation [98]. Therefore, rather than being a direct CK2 inhibitor, CIGB-300 was selected for interfering with CK2 phosphorylation by interacting with one of its targets. Consistently, it was shown that treatment with CIGB-300 induces apoptosis in several tumour cell lines (Table 1). Furthermore CIGB-300 was shown to significantly reduce tumour growth in syngeneic C57BL6 mice implanted with TC-1 lung epithelial tumour [98]. This initial observation stimulated further studies aimed at identifying the protein(s) targeted by CIGB-300 in vivo. Pull-down experiments in NCI-H82 cells (small cell lung cancer) identified 20 proteins: two known CK2 substrates, NPM1 and nucleolin as well as several ribosomal proteins. Subsequent experiments demonstrated that CIGB-300 directly binds NPM1 and not nucleolin *in vivo,* and suggested that nucleolin and ribosomal proteins pull-down may be mediated by NPM1 [99]. Using an *in vitro* CK2 phosphorylation assay, it was shown that NPM1 phosphorylation inhibition is due to direct binding of CIGB-300 to NPM1. In particular, CIGB-300 inhibits CK2 phosphorilation of NPM1 at Ser125 and rapidly co-localize with NPM1 at nucleoli after administration (Figure 3). Nucleolar disassembly was also observed after treatment with CIGB-300 as monitored by the rapid nucleoplasmic redistribution of both NPM1 and fibrillarin. CK2 was previously shown to be a master regulator of ribosome biogenesis [100]. Furthermore, CK2 inhibition by DRB was shown to trigger nucleolar breakdown and interfere with ribosome biogenesis. Notably similar effects were also observed by mutating NPM1 Ser125 [101]. These observations suggest that the activity of CK2 as a master regulator of nucleolar assembly and ribogenesis is operated through NPM1 as a downstream effector. CK2 mediated phosphorylation of NPM1 has also been shown to regulate its chaperone activity [102]. NPM1 inhibition might therefore interfere with the proper folding of ribosomal proteins, their correct loading on the nascent ribosome, but also with histone assembly at nucleolar rDNA. Even though the precise mechanism connecting nucleolar breakdown to apoptosis is not clear, data obtained with CIGB-300 support the idea that NPM1 phosphorylation may be a target for cancer treatment in those tumours were high levels of NPM1 are present. CIGB-300 exerts a broad antiproliferative effect on cell lines derived from breast, cervical, lung, colon, prostate cancer and chronic lymphocytic leukemia (CLL) while a robust antitumour effect was also observed *in vivo* in mouse models of cervical and lung cancer as well as CLL [98, 103, 104]. Recently it was also observed that the concomitant administration of CIGB-300 and drugs like cisplatin, paclitaxel, doxorubicin or 5-fluorouracil cisplatin gives rise to synergic chemotherapeutic effects in lung and cervical cancer models [105].

In clinical research, CIGB-300 has been established to provide some benefits and to be safe and tolerable by local injection in cervical malignancies [106]. More recently, another phase I clinical study on patients with stage 1B2/II cervical cancer allowed the estimation of the maximum tolerated dose (MTD) and the pharmacokinetics/biodistribution profiles for CIGB-300 following local administration [107]. Collectively these data make of CIGB-300 the NPM1-targeting drug that has been more deeply investigated so far.

## AVRAINVILLAMIDE

(+)-avrainvillamide is an alkaloid (hereafter avrainvillamide; Figure 2C), firstly isolated from a marine fungal strain of *Aspergillus sp*. and initially shown to display antiproliferative activity on a panel of cancer cell lines [108]. In 2007 it was reported that avrainvillamide was able to form tight complexes with NPM1 *in vivo* [109]. Avrainvillamide is thought to act as an electrophile centre subjected to the nucleophilic addition of a sulphur group to its unsaturated nitrone moiety. Consistently, the NPM1-avrainvillamide complex was disrupted by iodoacetamide treatment. Binding assays on site-directed mutants revealed that avrainvillamide binds NPM1 primarily by alkylating Cys275, which is located in helix H2 of the C-terminal three-helix bundle (Figure 1 and Figure 3). This makes this alkaloid the first and only small molecule inhibitor known to directly bind the NPM1 C-terminal DNA-binding domain (Table 1). Cellular studies reported that HeLa S3 cells transfected with a siRNA targeting NPM1 exhibited enhanced sensitivity to avrainvillamide, providing a correlation between the antiproliferative effects of avrainvillamide and levels of NPM1. A following recent paper thoroughly investigated the effect of avrainvillamide-NPM1 association in AML [110]. It was shown that avrainvillamide binds both NPM1wt and NPM1c+ in vitro, with increased efficiency for the latter, probably due to its unfolded C-terminal domain. The effect of avrainvillamide on NPM1 cellular localization was studied both in cells carrying wild-type and predominantly nucleolar NPM1 (OCI-AML2) and in cells carrying heterozygous NPM1 mutation and the NPM1c+ phenotype (OCI-AML3). Treatment with sublethal doses of avrainvillamide (250 nM) was not effective in displacing NPM1 from nucleoli in OCI-AML2 cells. Conversely, NPM1c+ was observed to partially relocalize in nucleoli when OCI-AML3 cells were challenged with the same dose of avrainvillamide. Higher doses of avrainvillamide (4 μM) caused an apoptotic phenotype in both cell lines with condensed or fragmented nuclei. The cellular localization of NPM1 was however markedly different in the two cell lines. While NPM1 was displaced from nucleoli in OCI-AML2 apoptotic cells, it appeared to co-localize with the highly condensed nuclei of OCI-AML3 cells. Interestingly, the nucleolar relocalization effect of avrainvillamide on the mutant form of NPM1 was confirmed by transfecting cells with an eGFP-NPM1c+ construct. Conversely, treatment with the Crm1-exportin1 inhibitor leptomycin, resulted in the nucleoplasmic but not nucleolar accumulation of the eGFP-NPM1c+ construct. It was also shown that avrainvillamide binding to the mutated C-terminal domain at Cys275 does not result in domain refolding, which would have explained the re-localization effect. Therefore, avrainvillamide appears to act as a surrogate for the compromised nucleolar localization signal (NoLS) in NPM1c+ even in the presence of an unfolded C-terminal domain [110], through a mechanism that is currently unknown.

## TMPYP4

TmPyP4 (tetra-N-methyl-pyridyl porphyrin) (Figure 2D) is a positively charged porphyrin that binds G-quadruplex DNA with high affinity [111]. The potential of this molecule in cancer therapy has been recently investigated by different groups (Table 1) [111, 112, 113, 114].

NPM1 was recently recognized as a G-quadruplex binding protein and initially implicated in binding G-quadruplex sequences from the *SOD2* and *c-MYC* gene promoters [29, 42, 43]. These initial findings prompted investigations aimed at assessing whether there might be a link between DNA binding, especially at G-quadruplex regions, and nucleolar localization. Nucleoli are nuclear regions enriched in proteins and RNAs that are organized in correspondence of and around tandemly duplicated ribosomal DNA genes; bioinformatics and *in vitro* analysis of rDNA suggested that this gene contains as much as 14 different G-quadruplex regions in the non-template strand [115].

NPM1 was initially found to be associated to the rDNA throughout the whole gene [9] and further studies revealed that rDNA G-quadruplexes are effectively bound by NPM1 both *in vitro* and *in vivo* [116]. This activity is played by the C-terminal domain of the protein and depends critically on its folded state. It was shown that the C-terminal domain of the AML-linked NPM1 mutant A form, which lacks both Trp288 and Trp290, is unfolded and unable to bind rDNA G-quadruplexes, while the reinsertion of the two tryptophan residues was sufficient to restore the correct folding, G-quadruplex binding and nucleolar localization [40, 116]. Among the possible strategies for treating AML with mutated NPM1 a so-called “NPM1 nucleolar starvation” hypothesis was suggested [7, 71]. This is based on the observation that mutations are always heterozygous and a small but detectable fraction of the wild-type protein always resides in nucleoli of leukemic blasts, possibly because it is necessary for nucleolar functions such as ribogenesis and/or for the correct assembly of nucleoli. Therefore depriving nucleoli of NPM1 might cause nucleolar stress and induce an apoptotic response. A lower dosage of such NPM1-nucleoli depleting agents might be toxic for AML blasts with respect to healthy cells given their limited residual NPM1 pool. This hypothesis was tested using TmPyP4 as an investigational tool on OCI-AML2 leukemia cells and OCI-AML3 cells expressing NPM1c+. First it was shown, on both OCI-AML2 and OCI-AML3 cells, that TmPyP4 effectively displaces NPM1 from nucleoli, even at sub-cytotoxic amounts [116, 117]. Indeed, nucleolar protein content is heavily affected by TmPyP4 since two other important nucleolar proteins, i.e. nucleolin and fibrillarin, are also displaced. However, TmPyP4 was found to be more toxic on OCI-AML2 than on OCI-AML3 cells, both as a function of dose and time, despite the limited amount of nucleolar NPM1wt in OCI-AML3 cells as compared to OCI-AML2 [117]. Mechanistically, it was shown i) that OCI-AML3 cells contain reduced levels of both NPM1 and p53 as compared to OCI-AML2, ii) that levels of p53 in both cell lines decreased in the presence of TmPyP4 and iii) that p53 was activated, as monitored by elevation of p21 mRNA levels, in OCI-AML2 but not in OCI-AML3 cells [117]. This is possibly linked to p14ARF inhibition of HDM2 in OCI-AML2 cells following TmPyP4 treatment; an event that may not happen in OCI-AML3 cells since p14ARF is delocalized in the cytoplasm by NPM1c+ and degraded [78].

## ATRA/ARSENIC OXIDE, DEGUELIN AND (−)-EPIGALLOCATECHIN-3-GALLATE

All-trans-retinoic-acid (ATRA) (Figure 2E), alone or in combination with arsenic trioxide (ATO; As_2_O_3_), is currently the frontline treatment for acute promyelocytic leukemia (APL) harbouring the PML-RARα gene rearrangement [118, 119]. Since the use of these agents has provided excellent results in APL, the therapeutic potency of these two compounds either in association with one another or with other cytotoxic agents has been also recently investigated in the most common type of AML i.e. the one with mutated *NPM1* gene and NPM1c+ expression [120, 121]. Initially it was shown that ATO, but not ATRA, is effective in inducing apoptosis in AML cell lines carrying NPM1 mutation A (NPM1c+ phenotype), both OCI-AML3 and IMS-M2, as compared to AML cells with NPM1wt only. When ATO was combined with ATRA a striking cooperative action in inducing apoptosis was detected in OCI-AML3 cells. These results were also replicated using patients' primary blasts. The primary blasts carrying the concurrent *FLT3-ITD* mutation were more susceptible than those where *FLT3* was not affected and this is particularly important given the worse prognosis of these patients with respect to those carrying *NPM1* mutation only. Furthermore pre-treatment with ATO/ATRA was shown to greatly sensitize cells to daunorubicin, which is currently used in AML induction therapy [120]. Similar results were also obtained by El Hajj and coworkers [121]. ATO is known to target the promyelocitic leukemia protein (PML) and this was confirmed also in AML blasts carrying NPM1c+. Importantly it was also shown that ATO treatment results in decreased levels of NPM1c+ while levels of NPM1wt remain unchanged [120]. Surprisingly this effect was exerted also by ATRA and a synergistic behaviour in the combined treatment was observed. Pre-treatment with the proteasome inhibitor MG132 reverted this phenotype, suggesting that NPM1c+ disappearance after ATO/ATRA treatment is due to proteasomal degradation. Further experiments suggested that NPM1c+ specific degradation may be triggered by the oxidative stress induced by ATO. In particular, oxidation of NPM1c+ Cys288 (which replaces the tryptophan residue of the wild type protein) could be the reason that makes the mutant protein more susceptible to proteasomal-mediated degradation with respect to wild-type [120].

These results are very promising, especially if we consider that both ATRA and ATO are already in the clinic, but further studies are required to reach a thorough biochemical characterization of the effects played by these drugs in AML with *NPM1* mutations.

Therapeutic strategies focused on NPM1c+ specific degradation have also been invoked in studies centred on two natural compounds, i.e. deguelin (Figure 2F) and (−)-epigallocatechin-3-gallate (ECGT) (Figure 2G). Deguelin is a rotenoid molecule initially isolated from the African plant *Mundulea sericea* (Leguminosae) and later from other plants, which displayed potent and selective apoptotic and antiangiogenic effects on a variety of cancer cells (lung, prostate, gastric, and breast cancer) [122]. ECGT is the major polyphenol extracted from green tea, widely investigated for its antioxidant properties and for its possible effects in cancer prevention [123]. In both cases it was shown that treatment of OCI-AML3 cells with deguelin [124] and of IMS-M2 cells (also expressing NPM1c+) with ECGT [125], was effective in reducing NPM1c+ but not NPM1wt levels and in inducing apoptosis. NPM1 levels were instead not consistently affected when treating AML cell lines expressing NPM1wt only [124]. Nothing is known however about the mechanistic basis of NPM1c+ specific degradation triggered by either deguelin or ECGT treatment.

## NUCANT (N6L)

Recently, Destouches and collaborators have identified NPM1 as one of the targets of a promising anticancer compound, NucAnt 6L (N6L) [126]. N6L is a synthetic pseudopeptide consisting of a central 3_10_-helical template formed by a repetition of 6 pseudopeptide residues (Lys-Aib-Gly) in which, each of the six lysines is linked to other three pseudopeptides (Lys [CH_<2_N] Pro-Arg) (Figure 2H). This molecule has been shown to inhibit the formation of colonies of various cancer cell lines in soft agar assays with IC_50_ values ranging from 5 to 40 μM, and also to significantly inhibit invasion capacity in a metastatic melanoma cell model (Table 1) [127]. *In vivo*, upon administration, N6L rapidly localizes to tumour tissue in tumour-bearing mice inducing significant inhibition of tumour growth without evident toxicity [126].

The anticancer activity was initially ascribed to the interaction of N6L with nucleolin, however, further investigation also identified NPM1 as a N6L interaction partner. The interaction between NPM1 and N6L was deemed to be monophasic with a dissociation constant of 1 nM as inferred by Surface Plasmon Resonance (SPR) experiments with purified NPM1 [126]. *In vitro* experiments were conducted only using full-length NPM1 and therefore it is presently unknown which NPM1 domain is actually targeted by N6L. Since N6L is rich in lysine and arginine residues, it resembles some properties of positively charged peptides that also bind NPM1, such as those belonging to the HIV Rev and Tat proteins. Therefore N6L, through NPM1 binding, may interfere with some protein-protein associations played by the protein (Figure 3). Further studies are needed to dissect this interaction and to investigate the effect of the drug on cell lines carrying NPM1c+ mutation.

## YTR107

Recent evidences have shown that NPM1, when phosphorylated on threonine 199 (pT199NPM1), is a key component of the DNA double strand break (DSB) repair machinery [128]. In particular, in response to the formation of DNA DSBs, pT199NPM1 is recruited to the site of damage and binds ubiquitinated chromatin in a RNF8/RNF168 dependent manner, forming irradiation-induced foci (IRIF) that promote the repair of DNA DSBs [128]. The use of radiation therapy in cancer treatment is limited by the intrinsic resistance acquired by cancer cells through the increased efficacy of their DNA damage repair processes, thus the inhibition of DNA repair mechanisms in cancer cells exposed to ionizing radiation may represent a valid therapeutic approach and, in this context, NPM1 is a new promising target.

Sekhar and colleagues [129], through affinity-based solid-phase resin capture and liquid chromatography/tandem mass spectrometry (LC/MS-MS), have identified NPM1 as the biological target of YTR107, a potent radiosensitizing compound previously identified [130]. YTR107 ((Z) −5 - ((N-benzyl-1H-indol-3-yl) methylene) pyrimidine-2, 4, 6 (1H, 3H, 5H) trione) (Figure 2I) interferes with DNA damage repair mechanisms and is therefore capable of sensitizing to radiation different tumour cell lines, including HT29 colorectal adenocarcinoma cells, D54 glioblastoma cells, PANC1 pancreatic cancer cells, different breast cancer cell models and NSCLC cell lines (Table 1) [127]. Moreover, YTR107 significantly potentiated radiation-induced growth delay in HT29 tumour xenografts [129].

Evidence suggesting that the radiosensitization induced by YTR107 is mediated by NPM1 was also reported [131]. First it was shown that NPM1-null mouse embryonic fibroblasts (MEFs) but not NPM1-wt MEFs are deficient in DNA repair and are radiosensitive. Then, it was shown that treatment with YTR107 of NPM1wt MEFs, but not of NPM1-null MEFs, impaired the formation of pNPM1 irradiation-induced foci and triggered a significant dose-dependent radiosensitization [131]. YTR107 was also shown to bind to the N-terminal region of NPM1 (residues 1-122) responsible for protein oligomerization and to promote NPM1 monomerization (Figure 3) [131]. Very recently, the synthesis of YTR107 analogues with increased efficacy on several cell lines, including OCI-AML3, has also been reported [132].

### Strategies in NPM1 targeting

**Figure 4:**
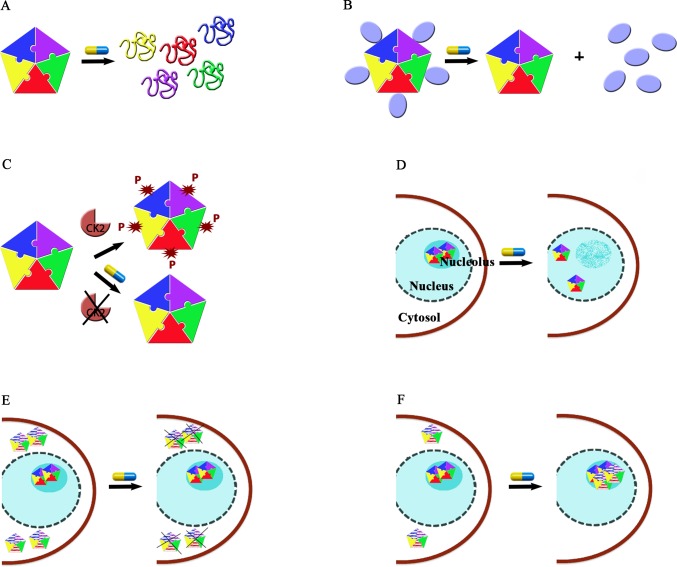
Strategies for NPM1 targeting **A.** Interfering with monomer-monomer interactions at the N-terminal domain would cause domain unfolding and the impairment of its functions. **B.** Interfering with the protein-protein interaction surface at the N-terminal domain might prevent most of the NPM1 anti-apoptotic activities. **C.** Interfering with NPM1 post-translational modifications such as CK2-mediated phosphorilation. **D.** Interfering with NPM1 C-terminal domain interactions with nucleic acids would result in nucleolar stress due to the dissociation of the protein from nucleoli (nucleolar starvation hypothesis). **E.** Selective degradation of NPM1c+ while leaving wild-type NPM1 unaffected may be pursued in AML with *NPM1* mutation. **F.** Pharmacological chaperone strategy aimed at refolding the mutated C-terminal domain in NPM1c+ would relocate the protein in nucleoli thus counteracting NPM1c+ anti-apoptotic activities. In panels E and F, mutated NPM1 is distinguished from wild-type NPM1 because represented with horizontal white and coloured lines.

The molecules that we have described above all bind NPM1 or interfere with specific functions played by the protein and, collectively, exemplify six different strategies that can be adopted in NPM1 targeting, summarized in Figure 4.

A first strategy consists in interfering with NPM1 oligomerization (Figure 4A). This effect is best achieved through molecules that target the NPM1 N-terminal domain dimerization surface. This is for instance the case of NSC348884 and, possibly, of YTR107, which were both shown to promote monomer formation, destabilizing the pentameric ring. Such molecules were tested against a significant panel of cancer cell lines where NPM1 is overexpressed and showed significant activity, especially in combination with other drugs (NSC34884) or radiation (YTR107). Not surprisingly, given the high hydrophobicity of the NPM1 dimerization surface, both drugs are poorly water-soluble (Figure 2A and 2I). This may constitute an obstacle for all drugs targeting this NPM1 surface that may be possibly overcome by the development of appropriate delivery systems such as nanocarriers.

A second strategy consists in interfering with the NPM1 protein-protein interaction network (Figure 4B). Data available in the literature [28, 90] and unpublished data from our laboratory, all suggest that protein epitopes enriched in positively charged residues are specifically recognized by the NPM1 N-terminal domain. Importantly, it is highly probable that NPM1 recognizes its protein partners' epitopes with the same surface. Therefore, targeting this surface would hamper several anti-apoptotic functions played by the protein simultaneously. This strategy is so far exemplified by the Rev-NLS peptide, which was effective against the Ras-3T3 cell line, also engrafted in mice, through a p53-dependent apoptotic response. Moreover, the pseudopeptide N6L was found to be effective against a large panel of cancer cell lines and structurally resembles NPM1 interacting peptides. Further design or optimization of molecules that display high affinity and specificity for the NPM1 protein interacting surface would be greatly facilitated if we had structures of the complexes between the NPM1 N-terminal domain and different protein partners.

A third strategy consists in interfering with NPM1 post-translational modifications (Figure 4C). This is exemplified by the cyclic peptide CIGB-300 that targets NPM1 Ser125 and prevents its phosphorylation by CK2, causing nucleolar stress and nucleolar breakdown followed by apoptotic cell death. CIGB300 has demonstrated pharmacological activity against a large panel of tumour cell lines where NPM1 is overexpressed and in mice xenografts. Its mechanism of action is unique among NPM1 interacting drugs however, since Ser125 is located just at the end of NPM1 N-terminal domain, it cannot be excluded that its action may also be that of impairing some NPM1 protein-protein association.

A fourth strategy is based on the so-called “nucleolar starvation hypothesis” [7, 72] according to which the selective displacement of NPM1 from nucleoli might cause nucleolar stress followed by apoptotic cell death (Figure 4D). TmPyP4 and Avrainvillamide actions may be both categorized under this strategy. Both compounds target the NPM1 C-terminal domain structure (avrainvillamide) or nucleic acid binding activity (TmPyP4) and are the only two drugs that are meant to interfere with this protein's domain activities. TmPyP4 effectively displaced NPM1 from nucleoli of AML cells and showed toxicity in a cell line where NPM1 is wild-type, due to p53 activation, while was relatively ineffective in a cell line carrying NPM1c+. Avrainvillamide exerted similar toxicity in the two cell lines but with a remarkable difference as regards to the NPM1 status: wild-type NPM1 was found displaced from nucleoli while NPM1c+ regained nucleolar localization (which let us categorize avrainvillamide also under a different strategy, see below). The NPM1 C-terminal domain surface involved in nucleic acid binding, and thus responsible for nucleolar localization, has been structurally elucidated and this offers a remarkable opportunity for the design of further molecules specifically aimed at interfering with this surface. Interestingly, while the nucleolar starvation strategy was initially proposed for the treatment of AML with NPM1c+, evidences obtained so far suggest that could it be effective also in tumours where wild-type NPM1 is overexpressed.

The strategies described above are relevant for targeting both solid tumours where NPM1 is overexpressed and AML with NPM1c+. The last two strategies instead may be considered specific for the latter.

The fifth strategy consists in the use of drugs causing the selective destruction of the mutated form of NPM1 (NPM1c+) while leaving wild-type NPM1 levels relatively unchanged (Figure 4E). Recent results suggest that the combined treatment with ATRA/ATO or the use of natural compounds like deguelin or EPGT may reach this goal. The underlying molecular mechanisms are totally unknown with respect to deguelin and EPGT. As to ATRA/ATO, it has been suggested that NPM1c+ might be more sensitive than wild-type NPM1 to the oxidative stress caused by ATO. This strategy is definitively worth further investigation especially in view of its high specificity for NPM1c+.

The final sixth strategy is the least investigated so far and consists in the so-called “pharmacological chaperone” approach [7]. In NPM1c+, the C-terminal domain of the protein is largely destabilized or totally unfolded due to the loss of Trp288 and Trp290. As a consequence the protein loses its nucleolar localization. According to this strategy any drug capable of refolding the mutated C-terminal domain should restore NPM1c+ nucleolar localization thus counteracting NPM1c+ cytosolic antiapoptotic activities (Figure 4F). Interestingly, avrainvillamide was shown to act as a surrogate of a pharmacological chaperone, being capable of inducing NPM1c+ nucleolar relocalization, but without refolding the protein's C-terminal domain.

Finally, always with reference to AML with *NPM1* mutations, it is worth mentioning an additional strategy consisting in the use of nucleolar stress inducers that already are in the clinic. For instance, promising results were obtained by treating patients with actinomycin D, a well known RNA polymerase I inhibitor [133].

## CONCLUSIONS

The studies we have reviewed here have shown a therapeutic potential for many molecules that interact with NPM1. Moreover, a striking synergy was observed in many cases when NPM1-targeting compounds were administered in combination with different chemotherapeutic agents or radiotherapy. This suggests that interfering with NPM1 status or functions may be a general way to sensitize cancer cells. Even though it cannot be excluded that, at least in some cases, cellular responses could be due not only to a direct effect on NPM1 and its interactors but also to indirect effects like those following DNA damage, it is clear from these studies that NPM1 targeting may be a powerful strategy for treating a number of tumours of diverse histological origin.

This is a field still in its infancy. Very few tests in animal models were performed and only two compounds (CIGB-300 and Nucant N6L) have entered clinical trials (Table 1). Indeed, many of the compounds we have reviewed here have been discovered or recognized for their effect on NPM1 only in the last two or three years. Furthermore, they not always display chemical features suitable for their development as drugs. Therefore, we anticipate that new molecules will be discovered, pursuing any of the different strategies that we delineated above.

One important issue that arises from the analysis of the available molecules is that, in some cases, their influence on the NPM1 status and localization is not immediately understandable on the basis of our current knowledge of NPM1 structural features. For instance, even though it is well established that the C-terminal domain of the protein contains the NoLS and therefore is responsible for NPM1 nucleolar localization, molecules targeting the NPM1 oligomerization surface at the N-terminal domain were equally able to displace the protein from nucleoli. Moreover, molecules targeting the central region caused loss of oligomerization, a property that is currently ascribed to the N-terminal domain. These and other data indicate the need for a better description of NPM1 structure and interactions. In particular the structures of the NPM1 N-terminal and C-terminal domains have been determined in isolation but nothing is known about the central domain and if, how and when the three domains interact with each other and structurally cooperate to enable NPM1 to fulfil its functions. Furthermore, even though NPM1 interacts with a plethora of different proteins, none of these interactions has been structurally characterized. If we aim at successfully targeting NPM1 for cancer treatment we will also need to address these important issues.
